# Hierarchical deep learning models using transfer learning for disease detection and classification based on small number of medical images

**DOI:** 10.1038/s41598-021-83503-7

**Published:** 2021-03-01

**Authors:** Guangzhou An, Masahiro Akiba, Kazuko Omodaka, Toru Nakazawa, Hideo Yokota

**Affiliations:** 1 R&D Division, Topcon Corporation, Tokyo, Japan; 2 Graduate School of System Informatics, Kobe University, Kobe, Japan; 3 Image Processing Research Team, RIKEN Center for Advanced Photonics, RIKEN, Wako, Japan; 4 Graduate School of Medicine, Tohoku University, Sendai, Japan

**Keywords:** Image processing, Machine learning, Eye diseases

## Abstract

Deep learning is being employed in disease detection and classification based on medical images for clinical decision making. It typically requires large amounts of labelled data; however, the sample size of such medical image datasets is generally small. This study proposes a novel training framework for building deep learning models of disease detection and classification with small datasets. Our approach is based on a hierarchical classification method where the healthy/disease information from the first model is effectively utilized to build subsequent models for classifying the disease into its sub-types via a transfer learning method. To improve accuracy, multiple input datasets were used, and a stacking ensembled method was employed for final classification. To demonstrate the method’s performance, a labelled dataset extracted from volumetric ophthalmic optical coherence tomography data for 156 healthy and 798 glaucoma eyes was used, in which glaucoma eyes were further labelled into four sub-types. The average weighted accuracy and Cohen’s kappa for three randomized test datasets were 0.839 and 0.809, respectively. Our approach outperformed the flat classification method by 9.7% using smaller training datasets. The results suggest that the framework can perform accurate classification with a small number of medical images.

## Introduction

Artificial intelligence (AI) has been applied in medical image classification via deep learning algorithms trained on massive amounts of supervised data^1,2^. Gulshan et al.^3^ used deep learning to create an algorithm for the automated detection of two ocular diseases in retinal fundus photographs based on a dataset of 128,175 retinal images. Rajpurkar et al.^4^ succeeded in classifying 14 different diseases using deep learning based on 112,000 chest X-rays. In addition to applications in disease detection, AI has also been implemented to assist decision making related to treatment. For instance, Esteva et al.^5^ used 130,000 dermatological photographs to build a deep learning model that outperformed human dermatologists in deciding treatment plans for patients with two types of skins cancer. Typically, the more the input data provided, the better a deep learning-based model performs^6^.

However, the supervised data available in the medical field is limited, since high level knowledge is required to prepare such data. Thus, there is a need for techniques for building deep learning models with small amounts of supervised data. Many deep learning techniques are being developed to overcome the obstacle of insufficient supervised data in the medical field. One approach is to synthetically increase the number of available samples for training deep learning models through data augmentation based on the geometrical transformation of images, or to mimic the distributions from which the images are sampled^1,7^. Another approach is the ‘not-so-supervised’ learning case, which includes semi-supervised, multi-instance, and transfer learning; among these, transfer learning has recently become the most popular^8^. Transfer learning, inspired by human thought processes, is a method in which model knowledge is effectively transferred across partially related or unrelated tasks to solve a new task with minimal retraining^9^. A study by Kermany, et al.^10^ demonstrated the competitive performance of deep learning models built with transfer learning in classifying normal eyes and eyes with three macular diseases using 4000 optical coherence tomography images.

A recent report stated that the doctor’s diagnostic decision is hierarchically structured, where a fairly broad initial list of potential diagnoses is then into fewer potential options^11^. In AI field, there is a similar efficient concept called the hierarchical classification method, in which hierarchically structured local classification models are built according to a predefined hierarchy^12^. This method achieved good classification performance not only in fine-grained natural image classification^13^, but also in medical image classification^14,15^. Although it is effective, there have been few studies to discuss the effectiveness of hierarchical classification to construct deep learning models with small amounts of data.

AI has been developed for supporting the diagnosis of various ophthalmologic diseases. Glaucoma is a leading cause of blindness worldwide, and glaucoma-related blindness is irreversible if not detected early and treated appropriately^16^. Glaucoma is regarded as a multifactorial disease, and some ophthalmologists suggest that treatment ought to be categorized by its cause^17^. A published guideline that defines four types of retinal optic discs based on their morphology^18^ was shown to be a useful addition to the determination of a proper treatment plan in glaucoma management^19–21^. This classification is difficult for doctors as it is based on subjective assessments of medical images^22^. Optical coherence tomography (OCT) is a popular imaging modality used in glaucoma diagnosis as it can capture cross-sectional images to form volumetric data of the retina. There have been several studies on diagnosing glaucoma using OCT data with AI^23–26^; most of these have aimed at automatic detection for classifying normal and glaucoma cases, whereas few have targeted glaucoma classification, which is difficult but crucial for proper treatment of glaucoma patients.

The purpose of this study was to propose a novel training framework for building deep learning models of disease detection and classification by using small datasets. Our approach is based on a hierarchical classification method, where the healthy/disease information from first model is effectively utilized to build subsequent models for classifying the disease into its sub-types using a transfer learning method. The effectiveness of the proposed training framework using a small OCT dataset is demonstrated to classify the glaucoma sub-types.

## Methods

### Dataset

In this study, images of 156 normal and 798 glaucomatous eyes were obtained from the Tohoku University Hospital database. This study adhered to the tenets of the Declaration of Helsinki, and the protocols were approved by the Clinical Research Ethics Committee of the Tohoku University Graduate School of Medicine (study 2014-1-805). Participants provided their written informed consent to participate in this study. The ethics committees approved this consent procedure, and all methods were carried out in accordance with relevant guidelines and regulations. These images were reviewed and labelled by two glaucoma specialists. The glaucomatous eyes were further classified into four sub-categories based on the optic disc morphology according to the definitions used in Nicolela classification as focally ischemic (FI), myopic glaucomatous (MY), generalized enlargement (GE), and senile sclerotic (SS) discs. Images with discordant classifications between the two glaucoma specialists were excluded. A total of 156 normal, 118 FI, 266 GE, 307 MY, and 107 SS eye images were used. The demographic data of the final subjects are presented in Supplementary Table S1.

**Figure 1:**
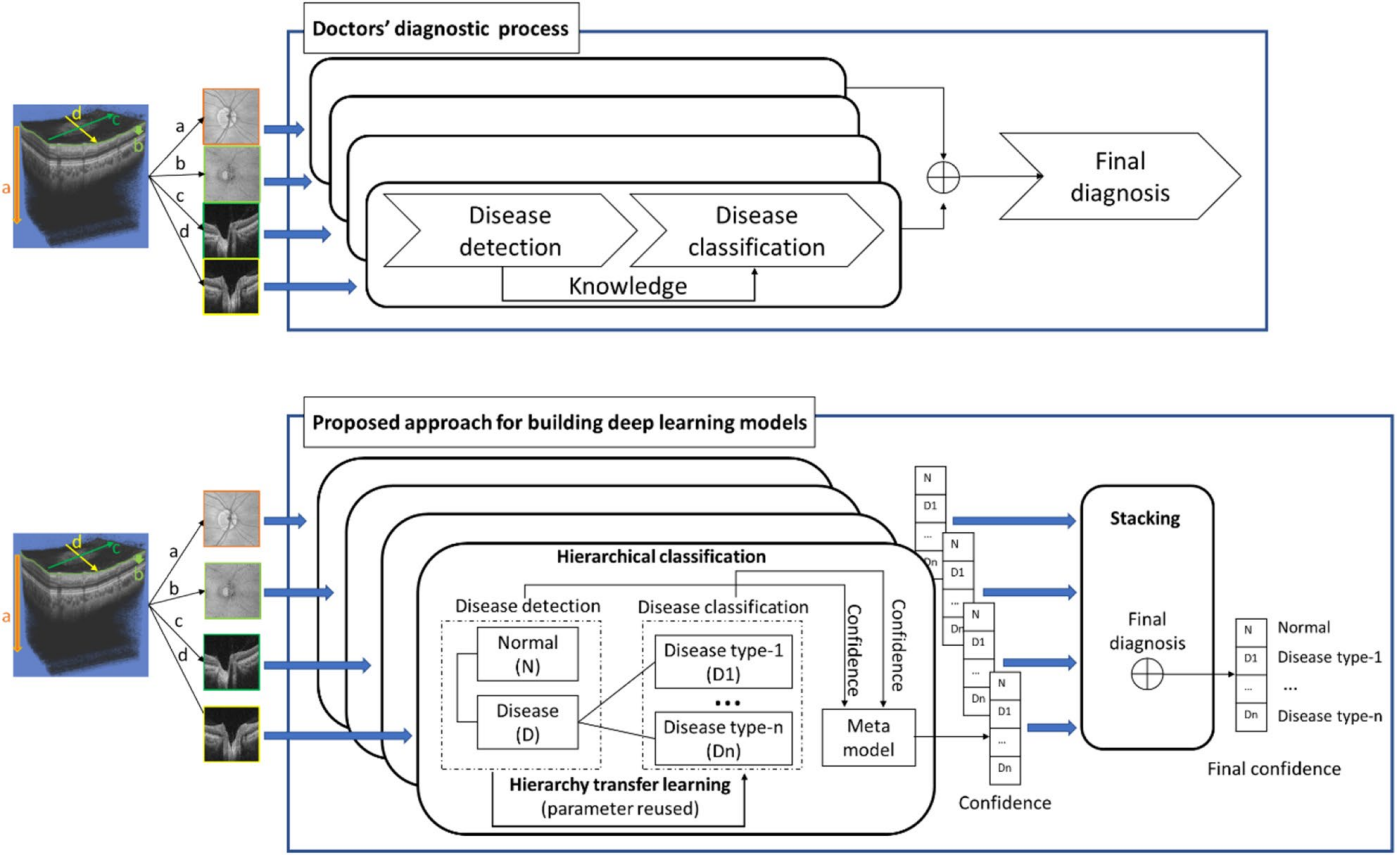
Overview of our main framework for building deep learning models. The top figure illustrates doctors’ diagnostic process. The bottom figure illustrates the building of deep learning models based on the analyzed characteristics of the doctors’ diagnostic process using three techniques: hierarchical classification, hierarchy transfer learning, and a stacking ensemble method.

**Figure 2:**
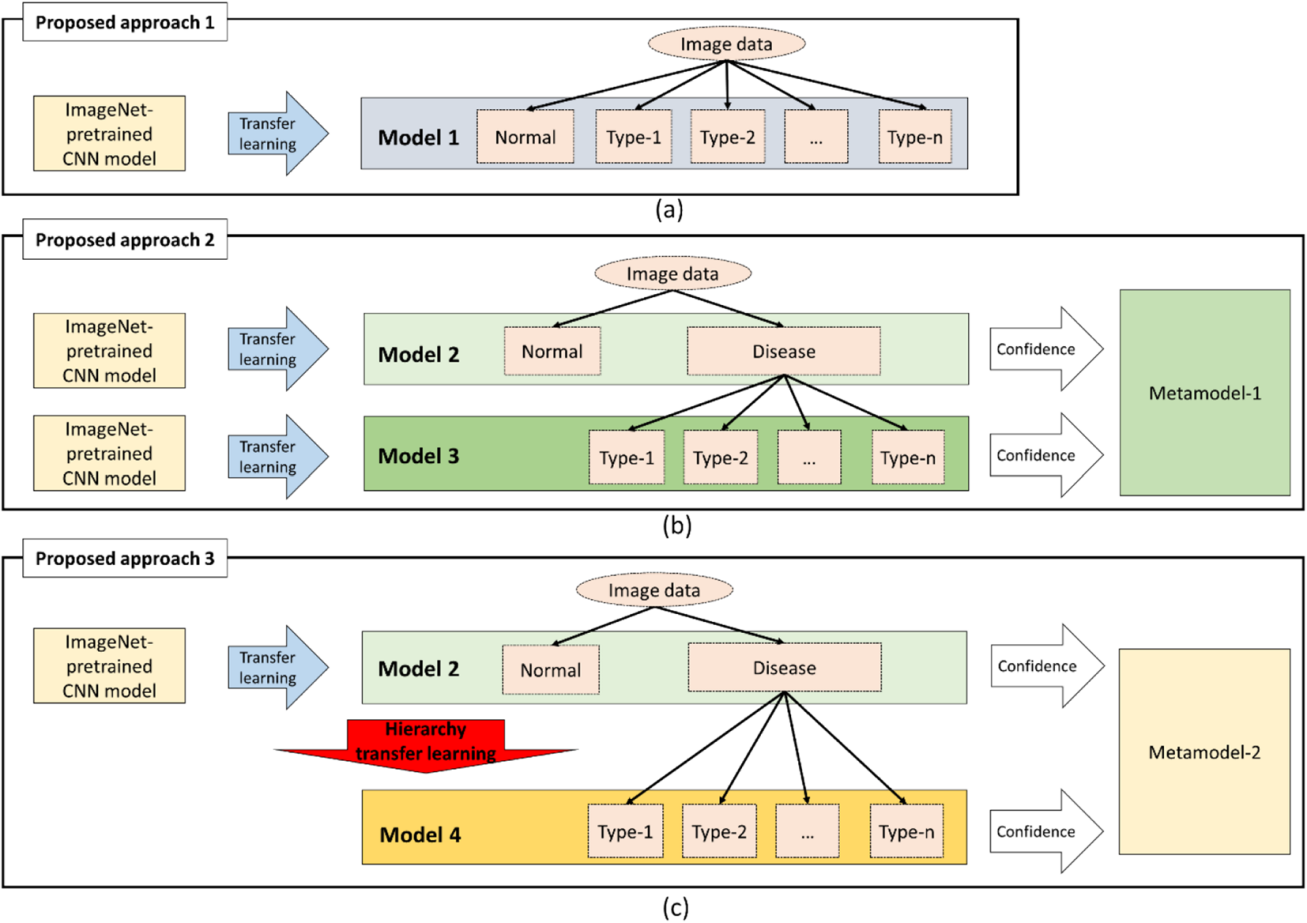
Proposed approaches for building deep learning models. Consider a classification problem (class number = *n* + 1) of normal and disease cases with *n* subtypes. **(a)** Proposed approach 1: flat classification is used to directly classify normal eyes and those with four subtypes of disease with transfer learning from an ImageNet-pretrained CNN model to create Model 1. **(b)** Proposed approach 2: hierarchical classification is used to create a low-level model (Model 2) for classifying normal versus disease cases and a high-level model (Model 3) for classifying subtypes of disease. Both models apply transfer learning from the ImageNet-pretrained CNN model. The confidence in a ‘normal’ result from Model 2 and the confidence in disease subtypes from Model 3 are concatenated to calculate the overall result from training Metamodel 1. **(c)** Proposed approach 3: hierarchical classification using hierarchy transfer learning between different-level models is used in the hierarchical classification model is. In contrast with proposed approach 2, the high-level model (Model 4) for classifying disease subtypes, transfer learning is from the low-level model (Model 2) instead of from the ImageNet-pretrained CNN model. The normal confidence from Model 2 and the disease subtype confidence from Model 4 are concatenated to train Metamodel 2 to calculate the overall result.

**Figure 3:**
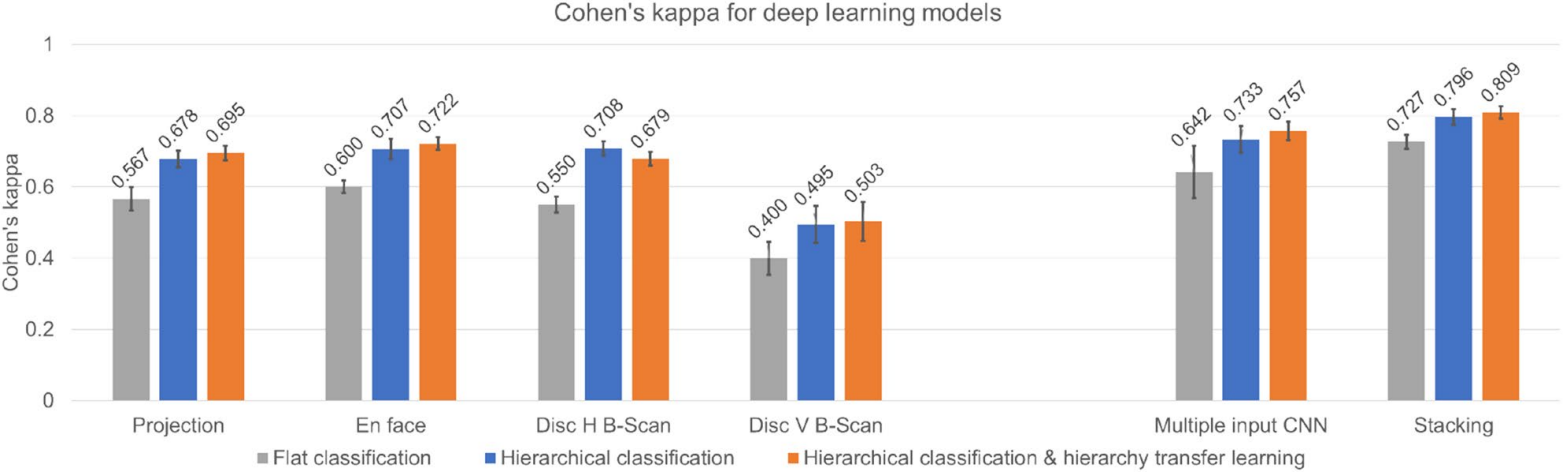
Classification performances of deep learning models built with the proposed approaches. The figure shows Cohen’s kappa with standard deviation error bars for the deep learning models for projection, en face, disc H B-scan, and disc V B-scan images and combination models using multiple-input CNN and stacking built with different proposed strategies.

These eye images were captured using swept source OCT (DRI OCT Triton), and a three-dimensional (3D) scan of the disc region (6.0 mm × 6.0 mm) was performed horizontally through 128 B-scans with 512 axial depth scans (A-scans) each. Four types of images, which the doctors used to analyze the 3D data for glaucoma diagnosis, were extracted from the 3D disc scan OCT data and used in our machine learning system: (1) projection images (image ‘a’ in Fig. 1); (2) en face images (image ‘b’ in Fig. 1); (3) horizontal B-scan OCT images crossing the disc center (image ‘c’ in Fig. 1; disc H B-scan); (4) vertical B-scan OCT images crossing the disc center (image ‘d’ in Fig. 1; disc V B-scan). Extraction and pre-processing details for these four types of images are provided in the subsection titled ‘Image Extraction and Pre-processing’ in the Supplementary Information.

### Proposed approach

First, we analyzed doctors’ diagnostic processes of disease detection and classification as follows (shown in the upper part of Fig. 1). (1) Doctors use limited information (e.g., a single type of medical image) to detect diseases. The determination of a treatment plan is difficult, as it requires disease classification based on complex symptoms, and it must be performed after disease detection (excluding normal images). (2) Doctors reuse the knowledge of classifying normal and diseased cases to classify diseased cases into subcategories. (3) For accurate diagnosis, doctors use multiple information sources (e.g., multiple types of medical images) to make optimal diagnostic decisions. Then, according to the analyzed characteristics of doctors’ diagnostic processes mentioned above, a novel two-step framework to build deep learning models for disease detection and classification was proposed to achieve high accuracy. In the first step, the model for classifying healthy and disease (disease detection) was built. In the second step, the model for disease classification was built by reusing parameters of the model for disease detection. This two-step framework was further extended to handle multiple input images by combining the machine learning models trained separately on single input images. In detail, we implement a training framework composing of three different methods including hierarchical classification, hierarchy transfer learning, and a stacking ensemble method. The first two components were used to build deep learning classification models with a single type of input image, and the last was used to combine these models. We applied hierarchical classification to separate the training steps of the model classifying normal versus disease cases and the model for disease classification. Hierarchy transfer learning from the model for classifying normal versus disease cases was used to build disease classification models based on the knowledge obtained from classifying normal and disease cases after the step of disease detection. For disease detection, we used transfer learning from a pretrained model on a large visual database (ImageNet) with more than 14 million natural images for visual object recognition. A metamodel was then used to combine the results of the model classifying normal versus disease cases and the model for disease classification using a single type of input images. Finally, a stacking ensemble method was used to combine the separate deep learning models to obtain the overall result (Fig. 1).

### Experiments and training

Two experiments were designed to verify our methods. One experiment compared the performance of different training approaches, whereas the other evaluated the applicability of the proposed approaches to small training data.

The first experiment was performed as described below by using the entire set of training data to compare classification performances among models trained using three proposed approaches (Fig. 2). We separately trained deep learning models for each type of extracted image from the 3D OCT data. A convolutional neural network (CNN), which can automatically create efficient image features for the classification, was used as the classifier^27^. Flat classification models were used to classify eyes as normal, FI, GE, MY, or SS directly with transfer learning from a deep learning model (a CNN) pretrained on the ImageNet dataset (ImageNet-pretrained CNN model) to create Model 1 (Fig. 2a, n = 4). We built a hierarchical classification model (Fig. 2b) applying transfer learning from the ImageNet-pretrained CNN model separately with a low-level model (Model 2 in Fig. 2b) for classifying normal versus glaucoma and a high-level model (Model 3 in Fig. 2b) for glaucoma classification (n = 4). Furthermore, the normal confidence from Model 2 and the confidence of FI, GE, MY, and SS from Model 3 were concatenated into a confidence vector length of 5. Then, a metamodel of the linear support vector machine (SVM) was trained using the confidence vector data with the supervised labels to combine the models in a cascaded manner^28^. As described in the section titled ‘Proposed Approach’, a hierarchical classification model applies transfer learning from the ImageNet-pretrained CNN model to create a low-level model for classifying normal versus glaucoma cases; then, it is hierarchically transferred to build a glaucoma classification model from the low-level model. Finally, a metamodel of the linear SVM was used to calculate the overall result (Fig. 2c).

A stacking ensemble method was used to combine the separately trained single-input image models. The confidence vector calculated by models trained with different single-input images was extracted and concatenated to train the superior metamodel via a linear SVM to combine the single-input models. For comparison with the stacking method, a multiple-input CNN with a direct four-image input was selected to handle multiple images and trained with the three proposed approaches (Fig. 2). A metamodel was also used to combine the results of different-level models to calculate the final classification result.

The second experiment was designed to evaluate the deep learning models’ applicability to small amounts of training data built using the proposed approach by using partial training data. Partial training datasets were created using a stratified random sampling strategy using different percentages of the entire training data (25.0%, 37.5%, 50.0%, 62.5%, 75.0%, 87.5%, and 100.0%). With the different training datasets, deep learning models were built with the proposed approaches (Fig. 2) using one type of input image (projection image) and a combination model via a stacking ensemble method for different single-input models.

In this study, we adopted the VGG-16 CNN architecture, which is widely used to solve image classification tasks^29^, for all the deep learning models and customized it as shown in the Model Architectures and Training Details in the Supplementary Information. In all the model training phases, data augmentation techniques were used to improve the models’ classification performance given the limited training data, as presented in the section titled ‘Model Architectures and Training Details’ in the Supplementary Information.

### Evaluation of classification models

The entire dataset was shuffled to create three different training (80%) and test (20%) datasets with a stratified sampling strategy. The training dataset was used to build the classification models, and the test dataset was used to evaluate the models. The average of the classification indexes for the three test datasets was used to evaluate the proposed approaches. Our dataset was not balanced in class distribution; thus, Cohen’s kappa and weighted accuracy were used to evaluate the deep learning models. Cohen's kappa coefficient is a statistic that measures the inter-rater reliability of qualitative items by considering the possibility of agreements occurring by chance^30^. The agreement thresholds used in this study were based on the following guideline: 0.61–0.80 corresponds to good agreement, and 0.81–1 corresponds to almost perfect agreement^30^. The weighted accuracy is computed by taking the average, over all the classes, of the fraction with the number of correctly predicted instances in that class, divided by the total number of instances in that class^31^. We used a paired t-test *P*-value (two-tailed) of 0.05 as the significance level to determine whether the mean difference between two sets of Cohen’s kappa was zero.

## Results

### Performance comparison of different training approaches

The Cohen’s kappa for the deep learning models of single-input images built with the flat classification strategy and transfer learning from the ImageNet-pretrained model (proposed approach-1 in Fig. 2a) were 0.567 for projection, 0.600 for en face, 0.550 for disc H B-scan, and 0.400 for disc V B-scan. Only the deep learning model using en face images achieved good performance with Cohen’s kappa over 0.6 (Cohen’s kappa: 0.600; weighted accuracy: 66.3%). The Cohen’s kappa for the deep learning models built using hierarchical classification without hierarchy transfer learning (proposed approach-2 in Fig. 2b) were 0.678 for projection, 0.707 for en face, 0.708 for disc H B-scan, and 0.495 for disc V B-scan. The deep learning models achieved good performance with Cohen’s kappa over 0.6 when using projection (Cohen’s kappa: 0.678; weighted accuracy: 75.5%), en face (Cohen’s kappa: 0.707; weighted accuracy: 74.6%), and disc H B-scan (Cohen’s kappa: 0.708; weighted accuracy: 77.5%) images. The deep learning models using hierarchical classification models provided significantly improved classification performance (P-values of the paired t-test: 0.027, 0.045, and 0.016, respectively) compared to models using the flat classification strategy for the same input images. The Cohen’s kappa for the deep learning models built with hierarchical classification and hierarchy transfer learning (proposed approach-3 in Fig. 2c) were 0.695 for projection, 0.722 for en face, 0.679 for disc H B-scan, and 0.503 for disc V B-scan. The deep learning models with the projection, en face, disc H B-scan as the input achieved good performance (projection (Cohen’s kappa: 0.695; weighted accuracy: 76.4%), en face (Cohen’s kappa: 0.722; weighted accuracy: 77.3%), and disc H B-scan (Cohen’s kappa: 0.679; weighted accuracy: 74.4%)). The deep learning models using hierarchical classification models provided significantly improved classification performance (P-values of the paired t-test: 0.014, 0.037, and 0.021, respectively) compared to models using the flat classification strategy for the same input images. There was no considerable difference between hierarchical classification with or without hierarchy transfer learning for all types of input images (left part of Fig. 3).

Our stacked method of four models that were built with transfer learning and pretrained on ImageNet provided satisfactory performance, with a Cohen’s kappa of 0.727 and weighted accuracy of 71.8%. The multiple-input CNN models achieved a Cohen’s kappa of 0.642 and weighted accuracy of 69.2% with four input images (right part of Fig. 3). The standard deviation of Cohen’s kappa for stacking was smaller than that of the multiple-input CNN trained with the same proposed approaches. The multiple-input CNN was not significantly higher than the single-input CNNs of projection images, en face images, and disc H B-scans. However, classification performance significantly improved with the stacking method for each single-input model (*P*-values of the paired t-test: 0.014, 0.022, 0.006, and 0.012 for projection, en face, disc H B-scan, and disc V B-scan images, respectively).

The hierarchical classification strategy with or without hierarchy transfer learning between the low-level model for classifying normal versus glaucoma and the high-level model for glaucoma classification showed significantly improved classification performance compared to flat classification (*P*-values of the paired t-test for the hierarchical classification strategy without hierarchy transfer learning were 0.001, 0.049, 0.008, and 0.013, and with hierarchy transfer learning were 0.047, 0.048, 0.030, and 0.031, for projection, en face, disc H B-scan, and disc V B-scan images, respectively, for both cases). There was no significant difference between the results of the hierarchical classification strategy with and without hierarchy transfer learning after applying the stacking ensemble method. Proposed approach-3 (hierarchical classification and hierarchy transfer learning with the stacking method) achieved the highest Cohen’s kappa, 0.809, and weighted accuracy, 83.9%.

### Applicability evaluation of the proposed approaches in small training data

**Figure 4:**
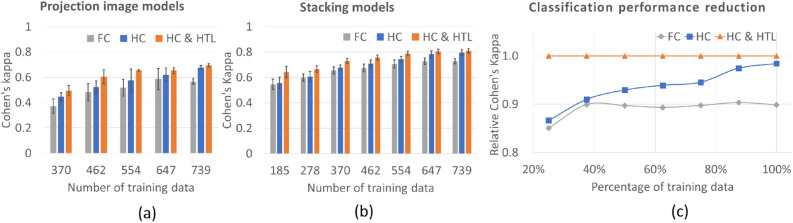
Performance change with different training dataset sizes. **(a)** Cohen’s kappa with standard deviation error bars for deep learning models trained with different training methods based on single-input (projection) images. **(b)** Cohen’s kappa with standard deviation error bars for deep learning models trained with different training methods based on all input images. **(c)** Calculated classification performance reduction for models using different training datasets and other training methods with stacking, with respect to Cohen’s kappa for the CNN model built with the proposed method of HC & HTL with stacking.

The performance change (as measured using Cohen’s kappa) provided by the deep learning classification models using projection input images was compared. All the models trained with flat classification (FC; Fig. 2a), hierarchical classification without hierarchy transfer learning (HC; Fig. 2b), and hierarchical classification with hierarchy transfer learning (HC & HTL; Fig. 2c) using a larger training dataset achieved an increase in Cohen’s kappa. Only HC and HC & HTL achieved satisfactory performance when using the entire training dataset. There was no significant difference between HC and FC in the case of a small training dataset. The HC & HTL strategy achieved a good Cohen’s kappa when using 62.5% of the entire training dataset (Fig. 4a).

The performance changes due to the stacked deep learning classification models using all the types of input images were compared. All the stacked models trained with FC, HC, and HC & HTL achieved a higher Cohen’s kappa (Table 1) compared to the single-input (projection) model using the same number of training datasets. With sufficient training data, all the models achieved convergence classification performance. With the same number of training datasets, the classification performance of HC & HTL was better than that of FC (Table 1, Fig. 4b; all *P*-values of paired t-tests < 0.05). The performance of the models built with the HC strategy was better than that of the models built with the FC strategy for large training datasets (more than 75.0% of the training dataset). However, there was no significant difference between HC and FC for a small training dataset (less than 75.0% of training dataset). The HC & HTL strategy achieved a fairly good Cohen’s kappa of 0.642 when using only 25.0% of the entire training dataset (Table 1, Fig. 4b). The performance reduction (relative Cohen’s kappa) for each model using each training dataset was calculated with reference to the Cohen’s kappa for the CNN model built with the HC & HTL strategy (Fig. 4c). The performance reduction of flat classification was greater than (1.000–0.903) × 100.0% = 9.7% compared to the use of the proposed method with the same training dataset (Table 1, Fig. 4c). The performance reduction of hierarchical classification was smaller than flat classification for each size of the training dataset, while the rate of performance reduction for hierarchical classification was larger than that with flat classification (Table 1, Fig. 4c).

**Table 1:** Performance change of stacking models with different training dataset sizes.

		Cohen's kappa			Relative Cohen's kappa		
Percent of total training data	Number of training data	FC	HC	HC & HTL	FC	HC	HC & HTL
25.0%	185	0.546	0.556	0.642	0.851	0.866	1.000
37.5%	278	0.600	0.606	0.666	0.899	0.910	1.000
50.0%	370	0.655	0.679	0.731	0.897	0.929	1.000
62.5%	462	0.675	0.710	0.756	0.893	0.939	1.000
75.0%	554	0.707	0.744	0.788	0.897	0.945	1.000
87.5%	647	0.727	0.784	0.805	0.903	0.975	1.000
100.0%	739	0.727	0.796	0.809	0.899	0.984	1.000

## Discussion

In this study, we built high-accuracy deep learning models for disease detection and classification. Experiments showed that the deep learning models trained with transfer learning from an ImageNet-pretrained CNN model with flat classification and data augmentation performed effectively in disease detection and classification on one type of input images (Fig. 3). The approach proposed by Kermany et al.^10^ is similar to proposed approach-1 (Fig. 2) as we adopted data augmentation for model training, which is useful in small datasets.

We proposed a two-step framework to build deep learning models for disease detection and classification based on the characteristics of the doctors’ diagnostic processes analyzed; the first step builds the model for disease detection, and the second step builds the model for disease classification by reusing parameters of the model for disease detection. Hierarchical classification was applied first for classifying diseased cases into subcategories after excluding the normal cases. The deep learning models built with hierarchical classification performed well and produced substantially improved results compared to flat classification for three types of input images (Fig. 3). Similar to our proposed approach-2 (Fig. 2), a previous study used hierarchy knowledge in disease classification and transfer learning from a pretrained model with a large natural image dataset and achieved a high accuracy^32^. We evaluated the applicability of this approach in relatively small datasets and found that it achieved higher performance than flat classification. As the variants for normal and diseased cases are different, we believe that hierarchically building models for disease detection and classification models can contribute to performance improvement. Moreover, we applied similar architectures for low-level and high-level models in the hierarchical classification model to further apply hierarchy transfer learning so that the parameters of models for classifying normal and diseased cases based on the analysis of the doctors’ diagnostic processes can be reused for classifying diseases into subcategories, which utilizes the knowledge of classifying normal and diseased cases. The combination of two methods (proposed approach-3) produced higher classification performance using smaller datasets than flat and hierarchical classification. While the knowledge of classifying normal and diseased cases is important to consider when classifying diseases into subcategories, it is believed that the hierarchy transfer learning method that attempts to reuse the parameters contributes to performance improvement when using relatively small training datasets.

We proposed two methods for handling multiple-input images based on doctors’ requirement of multiple images to make accurate diagnosis. As described in glaucoma guidelines, multiple sources of information, such as the patient’s medical history, visual acuity, assessment of the nerve fiber layer, and visual fields, are required to accurately assess early-stage glaucoma^33^. We extracted multiple useful images from one set of volumetric data to develop our deep learning models. The classification performance improved and was good for the multiple-input CNN and stacking methods. In comparison with the multiple-input CNN method, our stacking method considerably boosted the performance of four single-input CNNs with a small standard deviation. Doctors’ processes of making a diagnosis after asynchronously interpreting multiple images was imitated with the stacking method.

**Figure 5:**
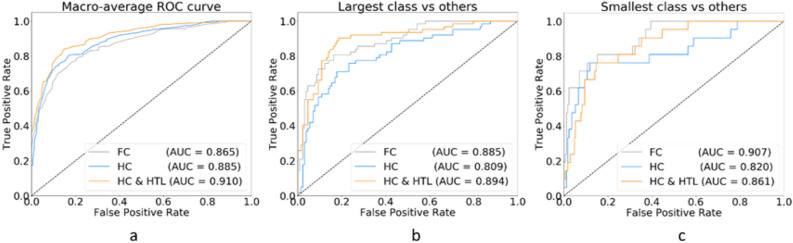
ROC curves for models built using a small training dataset. ROC curves for the stacked hierarchical classification model using hierarchy transfer learning: **(a)** macro-average ROC curve of all five classes, **(b)** ROC curves for the classification of the largest data class (MY) versus others, and **(c)** ROC curves for the classification of the smallest data class (SS) versus others.

Our proposed approach of hierarchical classification and hierarchy transfer learning (approach-3; HC & HTL) showed good compatibility with the stacking method and achieved higher classification performance than other proposed approaches. The classification performance was good even for small training datasets. The stacked deep learning models trained with three approaches (Fig. 2) achieved good classification performance using a small dataset (37.5% of the training dataset). The area under the receiver operating characteristic (ROC) curve (AUC) for each class, with the confidence calculated by three different proposed methods, was used to confirm the classification performance without a fixed threshold to predict. We found that our proposed approach of combining all the techniques achieved the best macro-average AUC (Fig. 5a), followed by approach-1 (FC) and approach-2 (HC), in recognizing normal, FI, and GE cases. For the largest class (MY) and smallest (SS) glaucoma-type classes in HC (Fig. 5b,c), the problem of imbalance affected performance, even when weighted loss was applied during training. The HC & HTL method provided the best classification performance of recognizing the largest class among the proposed approaches (Fig. 5b). In future studies, methods for overcoming the imbalance problem of the smallest class should be investigated.

A deep learning model for glaucoma detection and classification was built using the proposed framework that is implemented by hierarchical classification, hierarchy transfer learning, and the stacking ensemble method using different images, achieving a high Cohen’s kappa of 0.809. Based on the previous guideline of Cohen’s kappa^30^, this value implies a high performance of the decision support system for glaucoma clinical care. For comparison, we also conducted another diagnosis test. We randomly sampled images of 50 cases from the dataset used in this study, which were then classified into normal and glaucoma sub-categories by three medical ophthalmology interns. The average Cohen’s kappa of these classification results with supervised data was 0.408, lower than that of our deep learning model. The model can be used in diagnosis support of glaucoma by providing the confidence level for normal cases and each disc type, which would help doctors to select proper treatment plans according to the optic disc shape^20,34^.

Our model has a high potential for the automatic diagnosis of glaucoma. First, as more data are collected in future, the performance of deep learning models would improve. Next, many new deep learning techniques have been reported recently, such as deep learning architectures, which can boost the performance of deep learning models. Hopefully, deep learning models built with these new techniques and our approaches would achieve even better performance for glaucoma detection and classification. Finally, the images for which grading is difficult might be used in a semi-supervised classification manner to improve the performance of our deep learning models.

This study is novel in that it proposes a deep learning training framework using hierarchical classification and hierarchy transfer learning capable of building models that perform accurate disease detection and classification using a small number of images. The high potential of deploying deep learning models with a small number of datasets was demonstrated for practical diagnosis based on the characteristics of the clinical diagnostic processes.

## Conclusions

In this study, we proposed a novel two-step framework using hierarchy transfer learning to build deep learning models for disease detection and classification based on an analysis of the characteristics of doctors’ diagnostic processes. This training framework enabled the built deep learning models to achieve high accuracies and improved classification performances in comparison with models based on other training approaches. Moreover, the two-step framework was extended to handle multiple input images by combining models created with each type of input image with a stacking method to further improve the accuracy of doctors’ diagnoses. We showed that high accuracy and robustness can be achieved with a smaller number of training data using the proposed training framework. The effectiveness of the proposed approach and its applicability to diagnostic classification in medical images were demonstrated using a retinal image dataset relevant in glaucoma diagnosis. These results indicate the high potential of deploying deep learning models with a limited number of medical images for practical diagnoses through the analysis of the clinical diagnostic process.

## Supplementary Information

Supplementary Information.

## Data Availability

The images and diagnosis data used to support the findings of this study have not been made available because they are real clinical data from Tohoku University Hospital, and the patients’ rights to privacy should be protected, as it is possible to identify people from these data, but are available from the corresponding author on reasonable request.
