# Supplementary material for: Hierarchical deep learning models using transfer learning for disease detection and classification based on small number of medical images
Source: Sci Rep. 2021 Mar 1;11:4250. doi: 10.1038/s41598-021-83503-7 (PMC7921640; doi:10.1038/s41598-021-83503-7)
Supplement: Supplementary file 1 — Supplementary Information. [file 41598_2021_83503_MOESM1_ESM.docx]

Hierarchical deep learning models using transfer learning for disease detection and classification based on small number of medical images

Guangzhou An^1,2,3^, Masahiro Akiba^1,3^, Kazuko Omodaka^4^, Toru Nakazawa^3,4^ and Hideo Yokota^2,3*^

1 R&D Division, Topcon Corporation, Tokyo, Japan

2 Graduate School of System Informatics, Kobe University, Kobe, Japan

3 Image Processing Research Team, RIKEN Center for Advanced Photonics, RIKEN, Wako, Japan

4 Graduate School of Medicine, Tohoku University, Sendai, Japan

*email: hyokota@riken.jp

## **Supplementary Information**

**Supplementary Table S1. Demographic data of final dataset**

|  | Normal  n=156 | FI  n=118 | GE  n=266 | MY  n=307 | SS  n=107 |
| --- | --- | --- | --- | --- | --- |
| Gender  (male / female) | 52 / 104 | 33 / 85 | 126 / 140 | 142 / 165 | 78 / 29 |
| Mean deviation (dB) | - | -6.02±4.58 | -7.48±6.23 | -6.76±5.91 | -10.52±6.71 |
| Axial length (mm) | 24.08±1.07 | 24.47±1.25 | 24.50±1.37 | 25.87±1.27 | 25.18±1.33 |

## **Image extraction and preprocessing**

The disc centers were detected, and vitreous/inner limiting membrane boundaries were segmented automatically with Topcon’s commercial analysis software (FastMap V.10.13). The verification was performed by glaucoma specialists. Based on the position of the automatically detected disc centers and segmented vitreous/inner limiting membrane boundaries, four types of images were extracted from the 3D disc scan OCT data and used in our machine learning system. The four images. which the doctors used to analyse the 3D data for glaucoma diagnosis, are as follows: 1) projection images, integration performed across the entire image depth of 2.6 mm (image ‘a’ in Fig. 1); 2) en face images, integration performed across a fixed thickness of 52 μm (20 voxels) below the vitreous/inner limiting membrane boundary (image ‘b’ in Fig. 1); 3) horizontal B-scan OCT images crossing the disc center (image ‘c’ in Fig. 1; disc H B-scan); 4) vertical B-scan OCT images crossing the disc center (image ‘d’ in Fig. 1;disc V B-scan).. The region of a vertical length of 512 pixels was cropped from the disc H B-scan and disc V B-scan based on the average vitreous/inner limiting membrane’s axial position of the entire OCT data. The region of a vertical length of 512 pixels was cropped from the disc H B-scan and disc V B-scan based on the average vitreous/inner limiting membrane’s axial position of the entire OCT data. Finally, all the four images types were resized into 256 × 256 pixels and normalized to the range of 0 to 1.

## **Model architectures and training details**

For single-input CNN models, a CNN architecture VGG-16 was customized by adding batch normalization after each convolutional layer to accelerate training as the model classifier. Regarding the last two fully connected layers, the units of each layer were changed to 256 with a batch normalization layer and ReLu activation function.

For multiple-input CNN models, each input had the same feature extractor with the single-input CNN in our proposed approach of the weights pre-trained on ImageNet to extract features from the layer before the first fully connected layer. These were then concatenated and fed to newly created, two fully connected layers (both were 256 units with batch normalization layer and ReLu activation function).

In all classification models, only the unit number of the softmax layer was changed according to the class number of each classification task.

For all experiments, we applied the same training setup. Data augmentation techniques were used to improve the classification performance for limited training data, including horizontal flip, random rotation, and random shift. An epoch of 100 was used with a batch size of 32, the optimization method of stochastic gradient descent (SGD) with a learning rate of 10-3, and the weighted categorical cross entropy by the data size of each class as loss function. Finally, we selected the model with the minimum validation loss from 100 deep learning models with early stopping. Our experiments were performed using Python 3.6 on an Intel Xeon Gold 6130 @ 2.10 GHz of 32 GB of RAM with a Quadro GV100 (32 GB), using Keras 2.2.4 with TensorFlow 1.13.1.
